# Incidence of opioid‐induced constipation in Japanese patients with cancer pain: A prospective observational cohort study

**DOI:** 10.1002/cam4.2341

**Published:** 2019-06-24

**Authors:** Akihiro Tokoro, Hisao Imai, Soichi Fumita, Toshiyuki Harada, Toshio Noriyuki, Makio Gamoh, Yusaku Akashi, Hiroki Sato, Yoshiyuki Kizawa

**Affiliations:** ^1^ Department of Psychosomatic Internal Medicine and Supportive and Palliative Care Team National Hospital Organization Kinki‐Chuo Chest Medical Center Sakai Japan; ^2^ Division of Respiratory Medicine Gunma Prefectural Cancer Center Gunma Japan; ^3^ Department of Medical Oncology Kindai University Nara Hospital Nara Japan; ^4^ Center for Respiratory Diseases JCHO Hokkaido Hospital Sapporo Japan; ^5^ Department of Surgery Onomichi General Hospital, Onomichi Hiroshima Japan; ^6^ Department of Medical Oncology Osaki Citizen Hospital Miyagi Japan; ^7^ Medical Affairs Shionogi & Co., Ltd Osaka Japan; ^8^ Department of Palliative Medicine Kobe University Graduate School of Medicine Kobe Japan

**Keywords:** cancer pain, opioid, opioid‐induced constipation, prophylactic, spontaneous bowel movements

## Abstract

This multicenter, prospective, observational cohort study assessed opioid induced constipation (OIC) in Japanese patients with cancer. Eligible patients had stable cancer and an ECOG PS of 0‐2. OIC incidence based on the Rome IV diagnostic criteria was determined by patient diary entries during the first 14 days of opioid therapy. The proportion of patients with OIC was calculated for each 1‐week period and the overall 2‐week study period. Secondary measurements of OIC included the Bowel Function Index (BFI) score (patient assessment administered by physician), spontaneous bowel movements (SBMs) per week (patient assessment), and physician assessments. Medication for constipation was allowed. Two hundred and twenty patients were enrolled. The mean morphine‐equivalent dose was 22 mg/day. By Rome IV criteria, the cumulative incidence of OIC was 56% (95% CI: 49.2%‐62.9%); week 1, 48% (95% CI: 40.8%‐54.6%); week 2, 37% (95% CI: 30.1%‐43.9%). The cumulative incidence of OIC was lower in patients who received prophylactic agents for constipation (48% [95% CI: 38.1%‐57.5%]) than in patients who did not (65% [95% CI: 55.0%‐74.2%]). The cumulative incidences of OIC were 59% (95% CI: 51.9%‐66.0%), 61% (95% CI: 54.3%‐68.1%), and 45% (95% CI: 38.0%‐51.8%) based on BFI scores, physician assessments, and SBM frequency, respectively. Frequency of BMs/week before starting opioids was the most influential factor for the occurrence of OIC. Utilization of prophylactic agents for constipation was associated with a modest effect on reducing the incidence of OIC. The incidences of OIC reported were variable depending on the diagnostic tool involved.

## 1 INTRODUCTION

Opioid analgesics are the mainstay treatment for moderate‐to‐severe cancer pain and have been reported to provide relief in many patients.^1^, ^2^ A Cochrane review of randomized clinical trials of morphine for cancer pain reported that greater than 90% of patients with cancer pain achieved “no worse than mild pain” (a score of ≤30 on a 100‐mm visual analog pain intensity scale) with morphine.^3^ Adverse events, such as opioid‐induced constipation (OIC), however, can limit the clinical benefits of opioids and result in suboptimal pain management.^4^, ^5^, ^6^

OIC is characterized by difficult‐to‐pass and hard stools, straining at defecation, and sensations of incomplete evacuation or anorectal obstruction after the initiation of opioid treatment.^4^, ^7^, ^8^ Clinical manifestations of OIC depend on the agonist activity of opioids at peripheral μ‐opioid receptors in the enteric nervous system of the gastrointestinal tract.^9^ Stimulation of these receptors can delay gastric emptying, prolong colonic transit time, alter anal sphincter tone, and inhibit defecation.^9^

The incidence of OIC in patients with cancer is not well established, with reported estimates ranging from as high as 97% to as low as 5%.^10^ The availability of effective medication for OIC may play a role in the observed variation in incidence. Laxatives such as magnesium oxide have been widely used as a prophylactic treatment for OIC in Japan,^11^ but these agents have limited efficacy because they do not target the underlying pathophysiology of OIC.^5^, ^12^, ^13^, ^14^ Several targeted drugs (eg, methylnatrexone, naloxegol) belonging to a class known as peripherally acting μ‐opioid‐receptor antagonists (PAMORAs) are approved in the US for the treatment of OIC.^15^, ^16^ Naldemedine, another PAMORA, is approved in the US and Japan for the treatment of OIC in patients with chronic non‐cancer pain, and in Japan for patients with cancer.^17^, ^18^ A likely reason for the varying incidence of OIC is the use of several diagnostic criteria for reporting OIC in clinical trials and cross‐sectional studies, including the frequency of bowel movements (BMs), physician assessments, and the Bowel Function Index (BFI).^4^, ^19^, ^20^ Recently, researchers have incorporated criteria for OIC into the Rome IV criteria for colorectal disorders (see Materials and Methods).^21^, ^22^ Briefly, the Rome IV diagnostic criteria include key new or worsening symptoms of OIC such as low frequency of spontaneous bowel movements (SBMs), straining during defecation, a sense of incomplete evacuation and/or anorectal blockage, and hard or lumpy stool consistency.^21^ Here, we report on a prospective, observational study in Japanese patients with cancer pain, with the primary aim of estimating OIC incidence using the Rome IV criteria after the initiation of analgesic opioid therapy.

## 2 MATERIALS AND METHODS

### 2.1 Study design

This study (UMIN000025864) was conducted at 28 medical institutions in Japan and was approved by relevant institutional review boards (National Hospital Organization Kinki‐Chuo Chest Medical Center IRB, 1180 naga‐sone cho, Kita‐ku, Sakai, Osaka, Japan). The study was conducted in compliance with the Declaration of Helsinki and Ethical Guidelines for Medical and Health Research Involving Human Subjects. Before entering the study, all patients provided written informed consent.

The study was a multicenter, prospective, observational, cohort investigation of the incidence of OIC in Japanese patients with cancer pain who were starting strong opioid therapy. Following the initiation of opioid analgesic medication, patients used a paper diary (handwritten entries for 14 days) to record their bowel habits. Patients evaluated items, including date and time of BMs, form of stool using the Bristol Stool Scale,^23^ presence or absence of the feeling of incomplete evacuation, degree of straining, and sensation of anorectal obstruction/blockage during BMs (rated on a scale from 0 [none] to 4 [very severe]).

### 2.2 Eligibility criteria

Eligible patients (either sex) were aged ≥20 years old and had cancer that was expected to be stable for the duration of the study. In addition, patients had an Eastern Cooperative Oncology Group performance status (ECOG PS) score of ≤2, required the initiation of opioid analgesics, and had no constipation (≥3 BMs during the 7 days prior to enrollment). Patients were excluded if they had: (a) any current or cured conditions that could affect gastrointestinal tract structure or function; (b) surgery, an intervention, or radiotherapy affecting gastrointestinal function within 28 days prior to enrollment through the end of the study period; or (c) disimpaction during the 7 days prior to enrollment through the end of the study period.

### 2.3 End points

The primary study end point was the incidence of OIC, defined as meeting one of the Rome IV OIC diagnostic criteria, except criterion 1e (following page), which involves manual maneuvers and cannot be differentiated clearly from fecal disimpaction, which was an exclusion criterion. OIC based on the Rome IV criteria is defined as “(1) new, or worsening, symptoms of constipation when initiating, changing, or increasing opioid therapy that must include two or more of the following: (a) straining during more than one‐fourth (25%) of defecations, (b) lumpy or hard stools (Bristol Stool Form Scale 1‐2) more than one‐fourth (25%) of defecations, (c) sensation of incomplete evacuation more than one‐fourth (25%) of defecations, (d) sensation of anorectal obstruction/blockage more than one‐fourth (25%) of defecations, (e) manual maneuvers to facilitate more than one‐fourth (25%) of defecations (eg, digital evacuation, support of the pelvic floor), (f) fewer than three SBMs per week; and (2) loose stools [that] are rarely present without the use of laxatives.”^21^

Secondary end points included the incidence of OIC based on the attending physician's diagnosis of OIC, occurrence of <3 SBMs per week,^24^, ^25^, ^26^, ^27^ in which SBM is defined as any BM, except for movements occurring within 24 hours after rescue use of laxative therapy, and a BFI score of ≥28.8^20^ (defined in the Supplementary Methods). Additional secondary end points were the incidence of OIC (Rome IV criteria) with or without prophylactic use of treatments, defined as a constipation agent initiated at the same time of opioid therapy, and the change in incidence of OIC by treatment with agents for constipation after onset of OIC.

### 2.4 Statistical analyses

A target sample of 220 patients was determined based on previously published data from the Japanese Study Group for the Relief of Opioid‐induced Gastrointestinal Dysfunction (J‐RIGID).^28^ Assuming an incidence of OIC of 40% or 50% and a total of 200 analyzable patients (accounting for ~10% patient dropout rate), the 95% confidence interval (CI) width would be 14.0% and 14.3%, respectively.

Two populations were defined for analysis. Full analysis set (FAS) 1 was defined as all enrolled patients, except those with ethical guideline violations, those with an observation period of <4 days, and those who did not take opioids during the observation period of ≥7 days. FAS 2 was defined as all patients in FAS 1 with an observation period of ≥7 days.

The primary study end point was evaluated each week in FAS 1. The incidence of OIC was calculated for week 1 based on data from days 1‐7 and for week 2 based on data from days 8‐14. The cumulative incidence of OIC was calculated as the percentage of patients given a diagnosis of OIC during week 1 or week 2. OIC was also evaluated during the first 2 weeks (days 1‐14) of opioid therapy in FAS 2.

Analyses of secondary end points of OIC incidence determined by physician diagnosis, SBMs per week, BFI score, and based on the presence or absence of prophylactic agent use were performed in FAS 1. Analysis of the change in incidence of OIC after the onset of OIC in week 1 was performed in FAS 2, with an observation period of ≥11 days. Among patients assessed as having OIC in week 1, the OIC reduction rate was calculated by the presence or absence of prophylactic agent use, separately for those with and without a newly initiated treatment regimen.

All statistical tests were performed on observed values with a two‐sided significance level of 0.05 without multiplicity considerations. The 95% CIs were calculated using the Clopper‐Pearson method. SAS software for Windows, Version 9.4 (SAS Institute Inc, Cary, NC) was used for data analyses.

## 3 RESULTS

### 3.1 Patients

**Figure 1:**
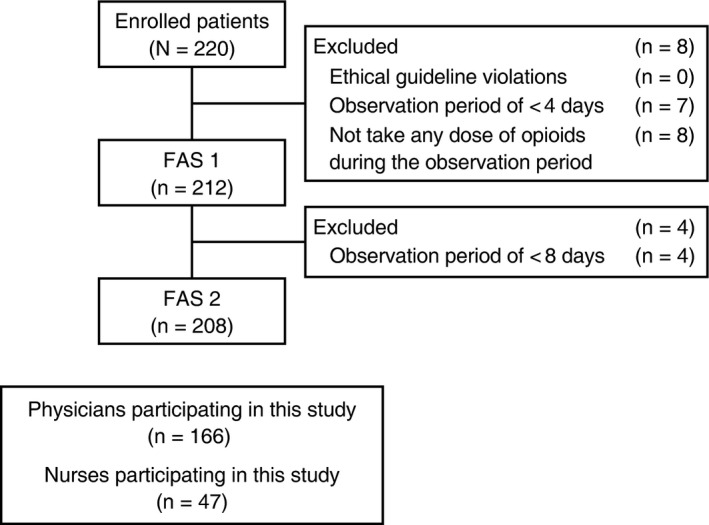
Patient disposition. FAS, full analysis set

Between 5 January 2017 and 31 January 2018, 220 patients were enrolled; 212 were included in FAS 1 and 208 were included in FAS 2 (Figure 1). Patient demographics and baseline clinical characteristics are summarized in Table 1. Most patients were male (68%), and the mean age was 69 years. The most common primary tumor type was in the lung (33%, FAS 1), and 50% of patients were receiving anticancer medication at the start of the study.

**Table 1:** Patient demographics and baseline clinical characteristics

Parameter	FAS 1 (n = 212)	FAS 2 (n = 208)
Sex, n (%)		
Male	145 (68)	141 (68)
Female	67 (32)	67 (32)
Mean age, years (SD)	69 (11)	69 (11)
Age group in years, n (%)		
≥20 to <40	0	0
≥40 to <50	13 (6)	13 (6)
≥50 to <65	48 (23)	48 (23)
≥65 to <75	84 (40)	84 (40)
≥75	67 (32)	63 (30)
BMs in past week, n (%)		
≥7/week	57 (27)	57 (27)
7/week	63 (30)	61 (29)
3‐6/week	92 (43)	90 (43)
<3/week	0	0
ECOG PS, n (%)		
0	51 (24)	50 (24)
1	121 (57)	119 (57)
2	40 (19)	39 (19)
Primary tumor type, n (%)		
Lung	69 (33)	67 (32)
Pancreas	26 (12)	25 (12)
Colon	25 (12)	25 (12)
Breast	14 (7)	14 (7)
Stomach	14 (7)	14 (7)
Esophagus	11 (5)	11 (5)
Prostate	10 (5)	9 (4)
Bladder	6 (3)	6 (3)
Other†	37 (17)	37 (18)
Metastasis, n (%)		
Yes	192 (91)	188 (90)
No	20 (9)	20 (10)
Anticancer medications, n (%)		
Yes	105 (50)	104 (50)
No	107 (50)	104 (50)
Admission status, n (%)		
Inpatient	115 (54)	111 (53)
Outpatient	97 (46)	97 (47)

Abbreviations: BM, bowel movement; ECOG PS, Eastern Cooperative Oncology Group performance status; FAS, full analysis set; SD, standard deviation.

^†^ Fewer than five patients for each tumor type.

Opioid analgesic regimens and the use of agents to treat constipation (eg, prophylactic treatment, regular‐use, and rescue‐use) are summarized in Table 2. During the study, patients received opioids at a mean morphine equivalent dose of 22 mg/d, and 51% of patients (FAS 1) received agents prophylactically for constipation. Magnesium oxide was the most commonly used prophylactic agent (46%), followed by sennosides (7%). Magnesium oxide was the most frequently used regular‐use agent for the management of OIC (65%), and sennosides were the most common rescue‐use agents (22%).

**Table 2:** Summary of opioid regimens and agents used to treat constipation during the study

Parameter	FAS 1 (n = 212)	FAS 2 (n = 208)
*Opioids*		
Mean dose, mg/d (SD)		
Overall	22 (15)	22 (15)
Regular‐use	19 (12)	20 (12)
Rescue‐use	6 (6)	6 (6)
Regular use, n (%)	193 (91)	189 (91)
Oxycodone	150 (71)	148 (71)
Morphine	16 (8)	14 (7)
Fentanyl	15 (7)	14 (7)
Tapentadol	10 (5)	10 (5)
Hydromorphone	13 (6)	13 (6)
Rescue use, n (%)	152 (72)	148 (71)
Oxycodone	118 (56)	116 (56)
Morphine	24 (11)	22 (11)
Fentanyl	2 (1)	2 (1)
Hydromorphone	9 (4)	9 (4)
*Agents for constipation*		
Prophylactic treatment, n (%)		
Received	109 (51)	108 (52)
Did not receive	103 (49)	100 (48)
Prophylactic agents, n (%)		
Magnesium oxide	97 (46)	96 (46)
Sennosides	14 (7)	14 (7)
Naldemedine	7 (3)	7 (3)
Senna	4 (2)	4 (2)
Lubiprostone	4 (2)	4 (2)
Sodium picosulfate	1 (0.5)	1 (0.5)
Others	2 (1)	2 (1)
Regular use, n (%)	152 (72)	149 (72)
Magnesium oxide	137 (65)	135 (65)
Naldemedine	29 (14)	28 (14)
Sennosides	18 (9)	18 (9)
Senna	5 (2)	5 (2)
Lubiprostone	5 (2)	5 (2)
Sodium picosulfate	1 (1)	1 (1)
Glycerin	0	0
Other	2 (1)	2 (1)
Rescue use, n (%)	101 (48)	99 (48)
Magnesium oxide	22 (10)	22 (11)
Naldemedine	1 (1)	1 (1)
Sennosides	46 (22)	45 (22)
Senna	4 (2)	4 (2)
Lubiprostone	4 (2)	4 (2)
Sodium picosulfate	30 (14)	29 (14)
Glycerin	13 (6)	12 (6)
Other	23 (11)	22 (11)

Abbreviations: FAS, full analysis set; SD, standard deviation.

### 3.2 Incidence of OIC by diagnostic criteria

**Figure 2:**
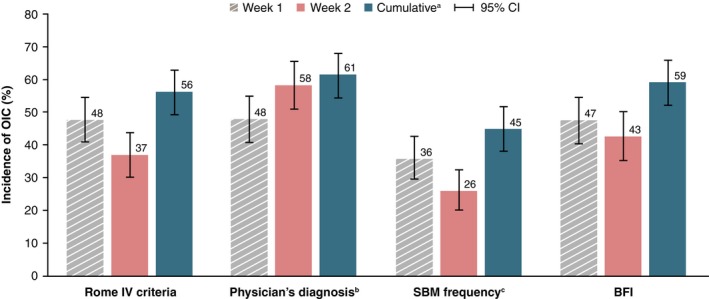
Incidence of OIC by diagnostic criteria (FAS 1). ^a^Patients were given a diagnosis of OIC in either week 1 or 2. ^b^Patients were given a diagnosis based on criteria for the presence or absence of OIC set by individual attending physicians. ^c^For this analysis, patients with <3 SBMs per week were given a diagnosis of OIC. SBMs were defined as any BM, except for movements occurring within 24 hours after rescue use of laxative therapy. BFI, bowel function index; CI, confidence interval; OIC, opioid‐induced constipation; FAS; full analysis set; SBM, spontaneous bowel movement

The cumulative incidence of OIC in either week 1 or week 2, based on the Rome IV diagnostic criteria, was 56% (119 of 212 patients; 95% CI: 49.2‐62.9) (Figure 2), and the incidence of OIC for the entire 2‐week study period was 44% (92 of 208 patients; 95% CI: 37.4‐51.3). In week 1, the incidence of OIC was 48% (101 of 212 patients; 95% CI: 40.8‐54.6), and in week 2, the incidence of OIC was 37% (74 of 201 patients; 95% CI: 30.1‐43.9).

The incidence of OIC varied depending on the diagnostic criteria used. Cumulative incidence was 61% (124 of 202 patients; 95% CI: 54.3‐68.1; Figure 2) based on the attending physician's diagnosis; 45% (95 of 212 patients; 95% CI: 38.0‐51.8) based on SBM frequency; and 59% (117 of 198 patients; 95% CI: 51.9‐66.0) based on BFI score.

### 3.3 Incidence of OIC by the use of agents for constipation

**Figure 3:**
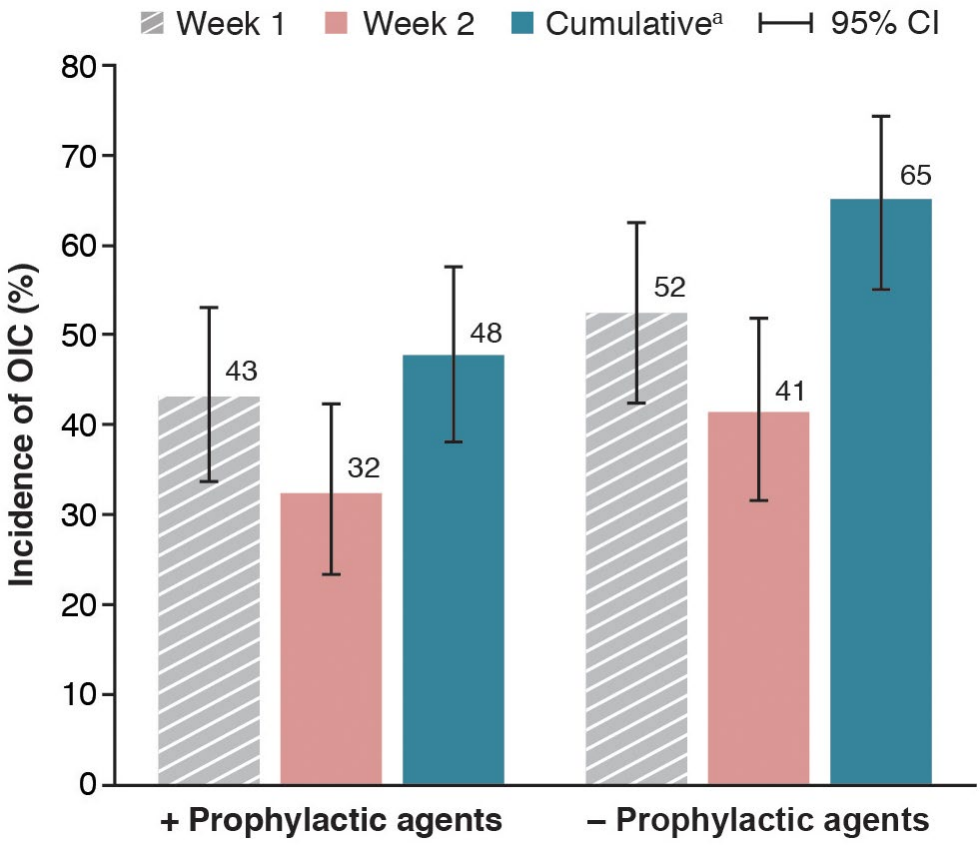
Cumulative incidence of OIC in patients who did or did not receive prophylactic agents for constipation (FAS 1). ^a^Patients were given a diagnosis of OIC in either week 1 or 2. CI, confidence interval; FAS, full analysis set; OIC, opioid‐induced constipation

The cumulative incidence of OIC by the Rome IV diagnostic criteria was lower in patients who received prophylactic agents for constipation (48% [52 of 109]; 95% CI: 38.1‐57.5) than in patients who received no prophylactic agents (65% [67 of 103]; 95% CI: 55.0‐74.2; Figure 3). Additional constipation medication that was given to patients who had an OIC diagnosis (defined by the Rome IV criteria during week 1 despite treatment with prophylactic agents for constipation) reduced the proportion of patients with OIC (ie, a relative reduction in OIC) by 34% (95% CI: 18.6‐53.2) (Supplementary Table 1). When no additional treatment was provided to patients who received prophylactic agents for constipation and were given a diagnosis of OIC during week 1, the relative reduction in the incidence of OIC was 36% (95% CI: 10.9‐69.2). Among patients who did not receive prophylactic agents for constipation, treatment after OIC diagnosis resulted in a 52% (95% CI: 30.6‐73.2) relative reduction in incidence of OIC. Patients who did not receive prophylactic agents for constipation or treatment following OIC diagnosis had a 41% (95% CI: 23.5‐61.1) relative reduction in OIC incidence.

### 3.4 Correlations between onset of OIC and patient background

Analyses of the cumulative incidence of OIC observed in week 1 or week 2 showed significant correlations between the onset of OIC and the baseline number of BMs per week (*P* = 0.0008), as well as the presence of comorbidities (*P* = 0.0205; Table 3). No significant correlations were observed for other baseline parameters, including sex, age, ECOG PS, tumor type, presence of metastasis, anticancer medications, and hospital admission status.

**Table 3:** Association between the onset of OIC and patient baseline characteristics (FAS 1)

Parameter	Cumulative Incidence of OIC, % (95% CI)	*χ* ^2^ test
Sex		*P* = 0.6321
Male	57 (48.8‐65.4)	
Female	54 (41.1‐66.0)	
Age group, years		*P* = 0.1587
≥40 to <50	77 (46.2‐95.0)	
≥50 to <65	65 (49.5‐77.8)	
≥65 to <75	54 (42.4‐64.5)	
≥75	49 (36.8‐61.8)	
BMs in past week		*P* = 0.0008
>7	37 (24.4‐50.7)	
7	56 (42.5‐68.1)	
3‐6	69 (58.0‐77.8)	
Comorbidities		*P* = 0.0205
Yes	52 (43.5‐59.6)	
No	70 (55.7‐81.7)	
ECOG PS		*P* = 0.5157
0	59 (44.2‐72.4)	
1	53 (43.6‐62.0)	
2	63 (45.8‐77.3)	
Primary tumor type		*P* = 0.5491
Lung	48 (35.6‐60.2)	
Pancreas	52 (31.3‐72.2)	
Colon	60 (38.7‐78.9)	
Breast	79 (49.2‐95.3)	
Stomach	71 (41.9‐91.6)	
Esophagus	60 (26.2‐87.8)	
Prostate	50 (18.7‐81.3)	
Bladder	50 (11.8‐88.2)	
Other	59 (42.1‐74.4)	
Metastasis		*P* = 0.7141
Yes	56 (48.4‐62.9)	
No	60 (36.1‐80.9)	
Anticancer medications		*P* = 0.1612
Yes	61 (50.9‐70.3)	
No	51 (41.5‐61.2)	
Admission status		*P* = 0.4964
Inpatient	58 (48.7‐67.4)	
Outpatient	54 (43.2‐63.8)	

Abbreviations: BM, bowel movement; CI, confidence interval; ECOG PS, Eastern Cooperative Oncology Group performance status; FAS, full analysis set; OIC, opioid‐induced constipation.

## 4 DISCUSSION

To the best of our knowledge, this is the first study to prospectively evaluate the incidence of OIC in Japanese patients with cancer pain. We found that approximately 50% of patients with cancer pain developed OIC within 2 weeks of initiating strong opioid therapy, despite receiving a low mean morphine‐equivalent dose (22 mg/d). These results indicate that even low‐dose opioids used to treat pain in patients with cancer are associated with an early onset of OIC. The reported incidence of OIC varied with the use of different diagnostic criteria and ranged from 45% (SBM frequency) to 61% (physicians’ diagnoses). This variability is not surprising given differences in the definition of OIC and potential biases associated with different diagnostic criteria, including the subjectivity of a physician's diagnosis. Our findings of higher OIC incidence based on physicians’ diagnoses compared with more objective measures is consistent with findings from the DYONISOS cross‐sectional survey of 520 patients with cancer pain who were receiving strong opioids.^19^ In that study, 86% of patients were diagnosed with OIC based on a physician's assessment, compared with 62% using the Knowles‐Eccersley‐Scott symptom score.^19^

In our study, we observed good agreement in the cumulative incidence of OIC using the Rome IV and BFI criteria (56% vs 59%, respectively). Good agreement was also observed using BFI and a visual analog scale in an evaluation of opioid bowel dysfunction patients receiving opioids for cancer or chronic noncancer pain.^29^ In that study, the reported OIC prevalence rate was 92% and 90%, respectively.^29^ Although the Rome IV criteria provided a more robust, detailed assessment of OIC compared with other diagnostic tools, we observed consistent results with BFI, suggesting that BFI could be a practical and convenient diagnostic tool for measuring OIC. This hypothesis is supported by conclusions from the American Academy of Pain Medicine (AAPM) 2015 consensus recommendations on initiating prescription therapies for OIC, which state that BFI is a “practical, validated, and responsive assessment tool that is clinically relevant in OIC.”^30^ BFI is particularly convenient because it consists of three items (“ease of defecation,” “feeling of incomplete bowel evacuation,” and “personal judgment of constipation”) that patients rate for the previous 7 days on a numerical analog scale of 0 (easy/no difficulty/not at all) to 100 (severe difficulty/very strong).^20^

This current study showed that the use of prophylactic treatment was associated with a reduction in the incidence of OIC (cumulative incidence of OIC: 48% among patients using prophylactic laxatives; 66% among patients not using prophylactic laxatives), which is consistent with findings from the retrospective J‐RIGID study. In the J‐RIGID study (N = 619), patients who were hospitalized and received prophylactic laxatives had significantly lower rates of OIC compared with those who did not receive prophylactic treatment (34% vs 55%, *P* < 0.001).^28^ These results support the current guidance from the European Consensus Group on Constipation in Palliative Care and the National Institute of Clinical Evidence suggesting prevention of OIC by prophylactic treatment with laxatives in patients receiving opioids.^31^, ^32^, ^33^ Nonetheless, in this study, a substantial proportion of patients receiving opioid analgesics experienced OIC despite prophylactic therapy. A potential explanation for this finding is that most prophylactic therapies given were laxatives and, therefore, limited by their lack of specificity for the etiology of OIC.^34^ A more robust study is needed to evaluate targeted prophylactic therapies for OIC (eg, PAMORAs) to determine if such treatments have a more pronounced effect in a prophylactic setting. Notably, in this study, the patients who did not receive prophylactic agents for constipation appeared to benefit most from postonset treatments.

Despite previous studies that reported OIC occurring more frequently in women and patients aged ≥ 50 years,^35^ we did not observe correlations between sex or age and incidence of OIC. Rather, these earlier findings^35^ may have been the result of higher rates of chronic constipation in these patient subgroups and may not have been specific to OIC.^36^ The current study excluded patients with chronic constipation, thereby eliminating potential confounding effects of sex‐ or age‐related risk associated with chronic constipation. Additionally, exclusion of patients with chronic constipation enabled a more accurate evaluation in the change in OIC after opioid administration. This study also excluded patients with higher ECOG PS, who typically experience decreased bowel movements and may have difficulty completing a patient diary, so as to eliminate potential confounding effects on constipation and reduce the likelihood of missing data in this patient group. The only patient characteristics that were significantly correlated with OIC onset were comorbidities and low frequency of SBMs prior to initiation of opioid therapy.

This study was limited by the relatively short 2‐week evaluation period from the start of opioid therapy. Additionally, the inclusion of OIC diagnosed by physician assessment may have introduced a potential source of bias. For this reason, a physician's diagnosis was designated as a secondary end point and the aim of this study was to evaluate OIC incidence rates using several different diagnostic criteria. Given the small sample size in this study, the effectiveness of specific PAMORAs was not evaluated.

## 5 CONCLUSIONS

The results of this study demonstrate that OIC can occur rapidly after the initiation of opioid therapy, even in patients who receive low doses of opioids. These data indicate that prophylactic treatment was associated with a decrease in the incidence of OIC. Although the current tools for diagnosing OIC result in variable rates of OIC incidence, rates observed using BFI scores were similar to those observed using the Rome IV criteria. Therefore, in agreement with the recent AAPM consensus recommendation, BFI may be an effective simple, practical, and reliable tool for assessing OIC in daily practice.

## CONFLICT OF INTERESTS

AT has received grants from Shionogi and Daiichi‐Sankyo and personal fees from Daiichi‐Sankyo, Mundipharma, Hisamitsu, Terumo, Sumitomo Dainippon, and Kracie. HI has received travel reimbursement from Shionogi. SF has received honoraria from Merck Serono, Ono Pharmaceutical Co., Bristol‐Myers Squibb, Takeda, and Bayer Yakuhin, and travel reimbursement from BeiGene. TH has received honoraria from Taiho Pharmaceutical, AstraZeneca KK, Boehringer Ingelheim, and Hisamitsu Pharmaceutical Co., Inc. TN has received personal fees from Shionogi, Taiho Pharmaceutical, and Chugai Pharmaceutical, and nonfinancial support from Shionogi. MG has received honoraria from Taiho Pharmaceutical, Chugai Pharmaceutical, Yakult Honsha, Ono Pharmaceutical, Takeda, Daiichi Sankyo, Nippon Kayaku, and Eli Lilly Japan. YA has received honoraria from AstraZeneca KK, Chugai Pharmaceutical Co., Ltd., MDS KK, Bristol‐Myers Squibb KK, Nippon Boehringer Ingelheim Co., Ltd., Pfizer Seiyaku KK, Taiho Pharmaceutical Co., Ltd., and Hisamitsu Pharmaceutical Co., Inc. HS is an employee of Shionogi & Co., Ltd. YK has received grants from Shionogi, Kyowa‐Kirin, and Terumo, and personal fees from Shionogi, Kyowa Kirin, Terumo, Johnson & Johnson, Daiichi‐Sankyo, Pfizer, Mundipharma, Chugai Pharmaceutical.

## Data Availability

The data that support the findings of this study are not publicly available.
